# Progress Toward Measles and Rubella Elimination — India, 2005–2021

**DOI:** 10.15585/mmwr.mm7150a1

**Published:** 2022-12-16

**Authors:** Ratnesh Murugan, Kristin VanderEnde, Veena Dhawan, Pradeep Haldar, Sourabh Chatterjee, Deepa Sharma, Kevisetuo Anthony Dzeyie, Subramanya Balakuntlam Pattabhiramaiah, Sudhir Khanal, Lucky Sangal, Sunil Bahl, Sukarma S.S. Tanwar, Michelle Morales, Ahmed M. Kassem

**Affiliations:** ^1^World Health Organization Country Office for India, New Delhi, India; ^2^Center for Global Health, Division of Global Health Protection, CDC India, New Delhi, India; ^3^Ministry of Health and Family Welfare, Government of India, New Delhi, India; ^4^JSI, New Delhi, India; ^5^Immunizations and Vaccines Development, World Health Organization South-East Asia Regional Office, New Delhi, India; ^6^Global Immunization Division, Center for Global Health, CDC.

In 2019, India, along with other countries in the World Health Organization (WHO) South-East Asia Region,* adopted the goal of measles and rubella elimination by 2023,^†^ a revision of the previous goal of measles elimination and control of rubella and congenital rubella syndrome (CRS) by 2020^§^ (*1*–*3*). During 2017–2021, India adopted a national strategic plan for measles and rubella elimination (*4*), introduced rubella-containing vaccine (RCV) into the routine immunization program, launched a nationwide measles-rubella supplementary immunization activity (SIA) catch-up campaign, transitioned from outbreak-based surveillance to case-based acute fever and rash surveillance, and more than doubled the number of laboratories in the measles-rubella network, from 13 to 27. Strategies included 1) achieving and maintaining high population immunity with at least 95% vaccination coverage by providing 2 doses of measles- and rubella-containing vaccines; 2) ensuring a sensitive and timely case-based measles, rubella and CRS surveillance system; 3) maintaining an accredited measles and rubella laboratory network; 4) ensuring adequate outbreak preparedness and rapid response to measles and rubella outbreaks; and 5) strengthening support and linkages to achieve these strategies, including planning and progress monitoring, advocacy, social mobilization and communication, identification and utilization of synergistic linkages of integrated program efforts, research, and development. This report describes India’s progress toward the elimination of measles and rubella during 2005–2021, with a focus on the years 2017–2021.^¶^ During 2005–2021, coverage with the first dose of a measles-containing vaccine (MCV) administered through routine immunization increased 31%, from 68% to 89%. During 2011–2021, coverage with a second MCV dose (MCV2) increased by 204%, from 27% to 82%. During 2017–2021, coverage with a first dose of RCV (RCV1) increased almost 14-fold, from 6% to 89%. More than 324 million children received a measles- and rubella-containing vaccine (MRCV) during measles-rubella SIAs completed in 34 (94%) of 36 states and union territories (states) during 2017–2019. During 2017–2021, annual measles incidence decreased 62%, from 10.4 to 4.0 cases per 1 million population, and rubella incidence decreased 48%, from 2.3 to 1.2 cases per 1 million population. India has made substantial progress toward measles and rubella elimination; however, urgent and intensified efforts are required to achieve measles and rubella elimination by 2023.

## Immunization Activities

**FIGURE 1:**
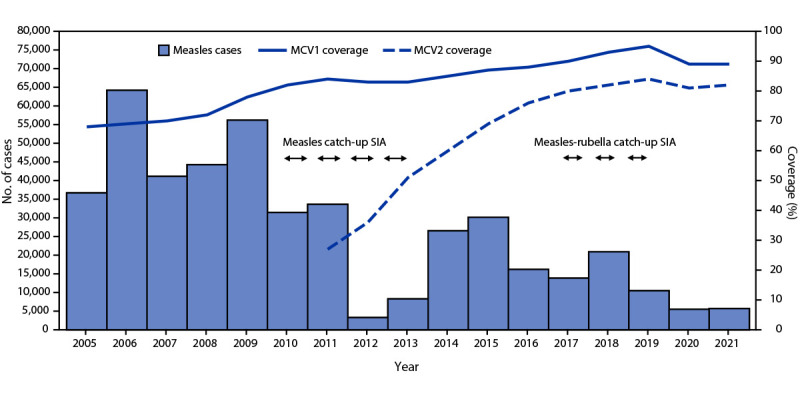
Number of reported measles cases,* estimated percentage of children who received their first and second doses of measles-containing vaccine,^†^ and supplementary immunization activities, by year^§^^,^^¶^ — India, 2005–2021 **Abbreviations:** MCV1 = first dose of measles-containing vaccine in routine immunization; MCV2 = second dose of measles-containing vaccine in routine immunization; SIA = supplementary immunization activity. * During 2005–2016, India’s Joint Reporting Form for measles and rubella included data from outbreak-based surveillance and additional sources. During 2017–2019, cases included data from outbreak-based surveillance and case-based measles and rubella surveillance. During 2019–2021, cases included data from cased-based measles and rubella surveillance and acute fever and rash surveillance. ^†^ Vaccination coverage data were from World Health Organization and UNICEF estimates of national immunization coverage; MCV1 was introduced into routine immunization in 1985, and MCV2 was introduced in 2011. ^§^ Measles catch-up SIA targeted children aged 9 months–10 years, implemented in three phases in 14 states: 2010–2011, 2011–2012, and 2012–2013. ^¶^ Measles-rubella catch-up SIA targeted children aged 9 months–15 years, conducted by state during 2017–2019 in 34 of 36 states and union territories.

**FIGURE 2:**
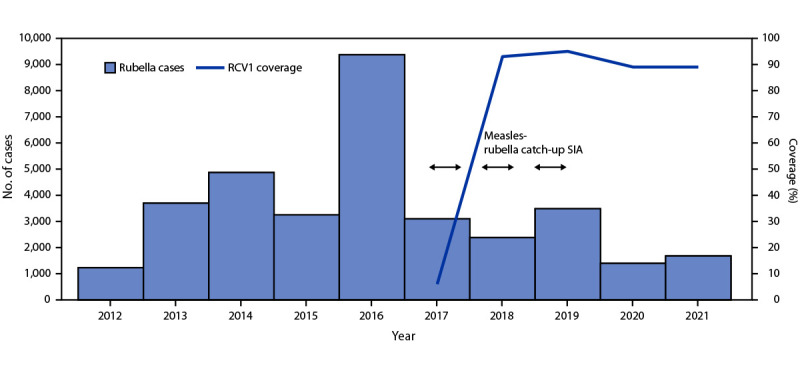
Number of reported rubella cases,* estimated percentage of children who received their first dose of rubella-containing vaccine,^†^ and supplementary immunization activities, by year,^§^ — India, 2012–2021 **Abbreviations:** RCV1 = first dose of rubella-containing vaccine in routine immunization; SIA = supplementary immunization activity. * During 2012–2016, India’s Joint Reporting Form for rubella cases included data from outbreak-based surveillance and additional sources. During 2017–2019, cases included data from outbreak-based surveillance and case-based measles and rubella surveillance. During 2019–2021, cases included data from cased-based measles and rubella surveillance and acute fever and rash surveillance. ^†^ Vaccination coverage data were from World Health Organization and UNICEF estimates of national immunization coverage; RCV1 was introduced into routine immunization in 2017. ^§^ Measles-rubella catch-up SIA targeted children aged 9 months–15 years, conducted by state during 2017–2019 in 34 of 36 states and union territories.

India has one of the world’s largest immunization programs, targeting a birth cohort of 27 million children annually (*5*). In 1985, coverage with a first dose of MCV (MCV1), administered at age 9–12 months, was introduced into the routine immunization program and MCV2, administered at age 16–24 months, was introduced in 2011. In 2017, India introduced RCV, and measles- and rubella-containing vaccine (MRCV) replaced MCV1 and MCV2 in the routine immunization schedule.** Administrative vaccination coverage (the number of vaccine doses administered divided by the estimated target population) is reported each year from all districts in India to the national immunization program, where data are aggregated and reported to WHO and UNICEF through the Joint Reporting Form. WHO and UNICEF use reported administrative coverage, country estimates, and vaccination coverage survey data to generate annual estimates of vaccination coverage through routine immunization services (*6*). Estimated MCV1 coverage increased 31%, from 68% in 2005 to 89% in 2021, and estimated MCV2 coverage increased 204%, from 27% in 2011 to 82% in 2021 (Table) (Figure 1). Estimated RCV1 coverage increased 1,383%, from 6% in 2017 to 89% in 2021 (Figure 2). The Fifth National Family Health Survey, conducted nationwide during 2019–2020, estimated the MCV1 coverage for children aged 12–23 months to be 88% compared with the 2005–2007 Third National Family Health Survey–estimated MCV1 coverage of 59% (*7*). Estimated coverage with the first MRCV dose (MRCV1) peaked at 95% in 2019 before the COVID-19 pandemic; coverage declined by 6 percentage points during the pandemic to 89% in 2020 and 2021. Similarly, the estimated MCV2 coverage declined from 84% in 2019 to 82% in 2021.

**TABLE:** Reported number of measles and rubella cases, by case classification, age group and vaccination status, and surveillance indicators — India, 2017–2021

Characteristic	No. (%)				
	2017	2018	2019	2020	2021
**Measles**					
**All cases, no.**	**13,854**	**20,925**	**10,485**	**5,497**	**5,697**
Laboratory-confirmed*	3,487 (25)	5,795 (28)	4,829 (46)	2,572 (47)	1,863 (33)
Epidemiologically linked^†^	9,569 (69)	13,470 (64)	3,291 (31)	629 (11)	448 (8)
Clinically compatible^§^	798 (6)	1,660 (8)	2,365 (23)	2,296 (42)	3,386 (59)
Incidence^¶^	10.4	15.4	7.7	3.9	4.0
Measles genotypes, no.	D4 (4), D8 (204)	D4 (1), D8 (333)	B3 (2), D4 (5), D8 (553)	B3 (4), D4 (64), D8 (510)	D8 (23)
**Age group of patients with laboratory-confirmed and epidemiologically linked measles**					
<9 mos	602 (5)	1.140 (6)	752 (9)	357 (11)	166 (7)
9 mos–4 yrs	5,255 (40)	7,579 (39)	2,840 (35)	1,371 (43)	981 (42)
5–9 yrs	5,144 (39)	7,449 (39)	2,177 (27)	743 (23)	552 (24)
10–14 yrs	1,466 (11)	1,944 (10)	960 (12)	295 (9)	291 (13)
≥15 yrs	589 (5)	1,153 (6)	1,391 (17)	435 (14)	321 (14)
Unknown or missing	NA	NA	NA	NA	NA
**MCV doses received by patients with laboratory-confirmed and epidemiologically linked measles**					
≥2	1,619 (12)	3,467 (18)	1,319 (16)	700 (22)	876 (38)
1	1,926 (15)	1,923 (10)	995 (12)	406 (13)	321 (14)
0	6,073 (47)	7,978 (41)	3,311 (41)	1,019 (32)	382 (17)
Unknown	3,438 (26)	5,897 (31)	2,495 (31)	1,076 (34)	732 (32)
**Rubella**					
**All cases, no.**	**3,097**	**2,381**	**3,487**	**1,397**	**1,681**
Laboratory-confirmed**	888 (29)	1,032 (43)	2,088 (60)	1,293 (93)	1,636 (97)
Epidemiologically linked^††^	2,209 (71)	1,349 (57)	1,399 (40)	104 (7)	45 (3)
Incidence^¶^	2.3	1.8	2.5	1.0	1.2
Rubella genotypes, no.	2B (9)	2B (9)	2B (19)	2B (6)	NA
**Age group of patients with laboratory-confirmed and epidemiologically linked rubella**					
<9 mos	115 (4)	92 (4)	169 (5)	109 (8)	82 (5)
9 mos–4 yrs	742 (24)	629 (26)	1,277 (37)	665 (48)	977 (58)
5–9 yrs	1,198 (39)	874 (37)	1,098 (32)	330 (24)	283 (17)
10–14 yrs	652 (21)	457 (19)	513 (15)	164 (12)	151 (9)
≥15 yrs	390 (13)	328 (14)	430 (12)	129 (9)	188 (11)
Unknown or missing	NA	1 (0)	NA	NA	NA
**RCV doses received by patients with laboratory-confirmed and epidemiologically linked rubella**					
≥2	64 (2)	108 (5)	187 (5)	157 (11)	345 (21)
1	74 (2)	52 (2)	489 (14)	342 (24)	464 (28)
0	1,801 (58)	1,323 (56)	1,882 (54)	608 (44)	524 (31)
Unknown	1,158 (37)	898 (38)	929 (27)	290 (21)	348 (21)
**Surveillance and program implementation**					
**States with case-based or fever and rash surveillance**					
Case-based surveillance^§§^	6 (17)	17 (47)	29 (81)	32 (89)	0 (0)
Fever and rash surveillance^¶¶^	0 (—)	4 (11)	4 (11)	4 (11)	36 (100)
**WHO-accredited measles and rubella laboratories, no.**	13	17	21	20	27
**States completing measles-rubella SIA**	10 (28)	26 (72)	34 (94)	34 (94)	34 (94)
**Surveillance performance indicators**					
No. of discarded NMNR cases***	3,581	7,196	14,514	11,039	25,654
No. of discarded NMNR cases per 100,000, national level (target ≥2)	0.3	0.5	1.1	0.8	1.8
Districts with NMNR discard rate ≥2	20 (3)	20 (3)	107 (15)	84 (11)	321 (42)
% of suspected cases adequately investigated^†††^ ≤48 hours after notification (target ≥80)	83	89	87	89	92
% of suspected cases with adequate specimens^§§§^ tested for measles and rubella in a proficient laboratory^¶¶¶^ (target ≥80)	100	100	100	100	99
% of samples tested ≤4 days after specimen receipt in laboratory (target ≥80)****	89	39	85	84	94
% of results received by program ≤4 days after specimen receipt (target ≥80)^††††^	67	22	47	53	72
% of weekly surveillance units reporting to national level on time (target ≥80)	92	92	94	94	93

**Abbreviations:** IgM = immunoglobulin M; MCV = measles-containing vaccine; NA = not applicable; NMNR = nonmeasles, nonrubella; RCV = rubella-containing vaccine; SIA = supplementary immunization activity; WHO = World Health Organization.

* Defined as a case that meets the suspected case definition and is laboratory-confirmed (serologically or virologically) as measles.

^†^ As a part of an outbreak investigation, additional suspected cases captured by online-listing forms but without specimens collected are epidemiologically linked if they are part of a laboratory-confirmed measles outbreak.

^§^ Defined as a suspected case for which no adequate laboratory specimen could be collected and is not epidemiologically linked to a laboratory-confirmed case of measles or rubella and not epidemiologically linked to another laboratory-confirmed communicable disease.

^¶^ Cases per 1 million population.

** Defined as a case that meets the suspected case definition and is laboratory-confirmed (serologically or virologically) as rubella.

^††^ As a part of an outbreak investigation, additional suspected cases captured by online-listing forms but without specimens collected are epidemiologically linked if they are part of a laboratory-confirmed rubella outbreak.

^§§^ A case-based surveillance suspected case is defined as illness in any person with fever and maculopapular (nonvesicular) rash and any one of cough, coryza (runny nose), or conjunctivitis (red eyes); or in any person in whom a clinician or health worker suspects measles infection.

^¶¶^ A fever and rash surveillance suspected case is defined as illness in any person with fever and maculopapular (nonvesicular) rash or in any person in whom a clinician or health worker suspects measles or rubella infection.

*** Suspected cases that have been investigated and discarded as nonmeasles and nonrubella by 1) laboratory result negative for measles and rubella through serum sample testing in a proficient laboratory and 2) no epidemiological linkage to another confirmed measles or rubella case.

^†††^ Suspected cases investigated ≤48 hours after notification.

^§§§^ Serum specimen collected ≤28 days (for serology) and throat swab/urine samples collected ≤7 days (for virology) after rash onset.

^¶¶¶^ A WHO-accredited laboratory that has an established quality assurance program or one with oversight by a WHO-accredited laboratory.

**** Samples tested for measles and rubella IgM ≤4 days after samples received in laboratory.

^††††^ Laboratory results for measles and rubella IgM received by program ≤4 days after sample receipt by laboratory.

During 2010–2013, India conducted a phased measles catch-up SIA for children aged 9 months–10 years in 14 states, vaccinating approximately 119 million children with MCV. In December 2014, India launched Mission Indradhanush (https://www.nhp.gov.in/mission-indradhanush1_pg) as a special immunization drive to vaccinate unvaccinated and partially vaccinated children aged <2 years living in selected districts. During 2015–2021, India completed four Mission Indradhanush rounds (periodic intensification of routine immunization activity), vaccinating approximately 39 million children who had previously missed any doses of vaccines provided through routine immunization, including measles and rubella (the latter during 2018–2021). During 2017–2019, India conducted measles-rubella SIAs in a phased manner in 34 (94%) of 36 states, vaccinating approximately 324 million children with MRCV. Of the two states that did not participate in the measles-rubella SIA, West Bengal has scheduled a measles-rubella SIA for early 2023, and Delhi has yet to confirm a date for the campaign.

## Surveillance Activities and Measles and Rubella Incidence

In 2005, India began using the WHO-supported acute flaccid paralysis polio surveillance platform for laboratory-supported measles and rubella outbreak-based surveillance in the state of Tamil Nadu. During the following 10 years, additional states began measles and rubella outbreak-based surveillance,^††^ which was implemented in all states by 2015, resulting in increased reporting of rubella cases in 2016 (Figure 2). During this period, India lowered the threshold for investigation of a suspected measles or rubella outbreak by 94%, from 20 cases per week in 2005 to five cases per 4 weeks in 2015. During 2005–2015, India’s Joint Reporting Form for measles and rubella cases included data from outbreak-based surveillance and additional sources. During 2017–2019, India transitioned from outbreak-based to case-based measles and rubella surveillance.^§§^ Furthermore, in 2021, after a pilot conducted in three states, India transitioned to case-based acute fever and rash surveillance^¶¶^ in all states (Table). To support this scale-up, the network of WHO-accredited laboratories expanded from three in 2005 to 13 in 2017; during 2017–2021, 14 additional laboratories were added to the network, for a total of 27.

Measles and rubella surveillance system indicators estimate sensitivity, timeliness, and function. During 2017–2021, the discarded nonmeasles and nonrubella cases rate,*** a measure of surveillance sensitivity, increased fivefold, from 0.30 to 1.81 per 100,000 population, and the percentage of districts with a discarded case rate ≥2 increased thirteenfold, from 3% to 42%. The timeliness of case investigations (≤48 hours of notification) improved from 83% in 2017 to 92% in 2021, and 100% of suspected cases with adequate specimens were tested in a WHO-accredited laboratory. In 2021, 94% of samples were tested ≤4 days of receipt by the laboratory; however, only 72% of laboratory results were submitted to the immunization program within 4 days of specimen receipt, potentially delaying public health action.

During 2017–2021, the incidence of measles decreased 62%, from 10.4 to 4.0 cases per million population, and the incidence of rubella declined 48%, from 2.3 to 1.2.^†††^ During this period, among the laboratory-confirmed and epidemiologically linked measles cases, 71% of patients had received no MCV doses or had an unknown vaccination history. Similarly, 81% of persons with laboratory-confirmed and epidemiologically linked rubella had received no RCV doses or had an unknown vaccination history. In 2021, among the laboratory-confirmed and epidemiologically linked cases, 42% of measles and 58% of rubella cases were reported in children aged 9 months–4 years.

Among isolates from patients during 2017–2021, measles virus genotypes detected and reported included B3, D4, and D8; D8 accounted for 1,623 (95%) of 1,703 isolates reported. Rubella virus genotype 2B was detected and reported from 43 patients during 2017–2021. However, genotype information is available for a small proportion of measles (3%) and rubella (0.4%) cases during this period.

## Discussion

During 2005–2021, India made substantial progress toward measles and rubella elimination. Through implementation of national and regional strategies, including Mission Indradhanush and two SIAs conducted in phases during several years each (2010–2013 and 2017–2019), to strengthen both routine and supplementary immunization, estimated MCV1, MCV2, and RCV1 coverage increased 31%, 204% and 1,383%, respectively. Reported measles and rubella incidence declined by 62% and 49%, respectively, during 2017–2021.

Despite this progress, India continues to face challenges in its goal to achieve measles and rubella elimination by 2023. During the COVID-19 pandemic, national routine MRCV1 coverage decreased from a peak of 95% in 2019 to 89% in 2021, and MCV2 coverage decreased from a peak of 84% (2019) to 82% (2021). In addition, the surveillance indicators demonstrated declines in sensitivity of measles and rubella surveillance from 2019 to 2020. India initiated various measures to mitigate the impact of the pandemic on immunization delivery and surveillance, including the dissemination of updated guidance for the continuation of immunization and surveillance, as well as state-level reviews to discuss challenges and track progress. During the second half of 2021, while continuing to respond to the COVID-19 pandemic, India trained approximately 240,000 persons in fever and rash surveillance at workshops throughout the country. In 2021, 42% of districts reached the surveillance performance target of two or more discarded nonmeasles and nonrubella cases per 100,000 population, an increase of approximately 280% from 11% of districts in 2020. To address challenges with data quality and laboratory and surveillance reporting delays, India is transitioning to a new, real-time, integrated Vaccine-Preventable Disease Surveillance Information Management System.

The findings in this report are subject to at least three limitations. First, coverage estimates are based on administrative data and might be inaccurate because of errors in recording doses administered or in estimating the target population. Second, surveillance data might underestimate actual disease incidence because surveillance sensitivity was low: children who had measles or rubella might not have been brought in for care and not all cases in patients who sought care might have been reported. Finally, given the small number of samples sequenced, genotype data might not reflect the predominant circulating genotypes.

In September 2022, recognizing the urgent and intensified work required to achieve measles and rubella elimination by 2023, India adopted a “Roadmap to Measles and Rubella Elimination in India by 2023” (*8*). Given subnational variations in immunization coverage and surveillance sensitivity, the roadmap includes an action plan to intensify and monitor progress toward measles and rubella elimination with a focus on district-level implementation, tracking, and program review. With an annual birth cohort of 27 million children in India, the measles and rubella elimination program represents a remarkable opportunity to prevent death and illness from these diseases.

**Summary**

**What is already known about this topic?**

In 2019, India adopted the goal of measles and rubella elimination by 2023, a revision of the goal of measles elimination and control of rubella and congenital rubella syndrome by 2020.

**What is added by this report?**

Estimated coverage with the first dose of a measles- and rubella-containing vaccine increased from 68% to 89% in 2021. Estimated coverage with the second dose of a measles-containing vaccine increased from 27% to 82% in 2021. During 2017–2021, measles and rubella incidence declined 62% and 48%, respectively.

**What are the implications for public health practice?**

India has made substantial progress toward measles and rubella elimination; urgent and intensified efforts are required to achieve elimination goals by 2023.

## Footnotes

* WHO South-East Asia Region includes 11 countries: Bangladesh, Bhutan, Burma, India, Indonesia, Maldives, Nepal, North Korea, Sri Lanka, Thailand, and Timor-Leste.

† Measles elimination is defined as the absence of endemic measles cases for ≥12 months in the presence of adequate surveillance. Rubella elimination is defined as the absence of endemic rubella cases for ≥12 months in the presence of adequate surveillance.

§ Rubella and congenital rubella syndrome control is defined as a 95% reduction in disease incidence from the 2013 level.

¶ Although India conducts sentinel site surveillance for CRS, national CRS incidence is not regularly reported. Modeled incidence of CRS in India has been published elsewhere and is beyond the scope of this report.

** India introduced a second RCV dose (RCV2) at the same time as RCV1, with MRCV replacing MCV in the routine schedule. However, there are no WHO and UNICEF estimates for RCV2 because RCV2 coverage data are not regularly reported.

†† Measles and rubella outbreak-based surveillance started in 2005 with outbreak definition of 20 cases per week. The outbreak threshold was changed in 2012 to 10 cases per week and in 2014 to five cases per week. In 2015, the outbreak threshold was changed to five cases per 4 weeks.

§§ For case-based measles and rubella surveillance, a suspected case was defined as a person with fever and maculopapular (nonvesicular) rash with cough, coryza or conjunctivitis, or any person in whom a clinician or health worker suspects measles infection.

¶¶ For case-based acute fever and rash surveillance, a suspected case was defined as a person with fever and maculopapular (nonvesicular) rash, or any person in whom a clinician or health worker suspects measles or rubella infection.

*** A discarded case is defined as a suspected case that has been investigated and determined to be neither measles nor rubella by 1) laboratory testing in a proficient laboratory or 2) epidemiologic investigation with no linkage to another confirmed measles or rubella case.

††† Measles cases include laboratory-confirmed, epidemiologically linked, or clinically compatible (a suspected case for which no adequate laboratory specimen could be collected) cases. Rubella cases include those that are laboratory-confirmed and epidemiologically linked.
